# The Influence of Bone Density on Stresses in the Periodontal Ligament During Orthodontic Movement—Finite Element Study on Innovative Model

**DOI:** 10.3390/ma18040776

**Published:** 2025-02-10

**Authors:** Anna Ewa Kuc, Kamil Sybilski, Michał Stankiewicz, Jacek Kotuła, Natalia Kuc, Grzegorz Hajduk, Jerzy Małachowski, Michał Sarul

**Affiliations:** 1 Department of Dentofacial Orthopedics and Orthodontics, Wroclaw Medical University, Krakowska 26, 50-425 Wroclaw, Poland; 2 Faculty of Mechanical Engineering, Military University of Technology, Sylwestra Kaliskiego 2 Street, 00-908 Warsaw, Poland; 3 Faculty of Medicine, Medical University of Bialystok, Jana Kilińskiego 1, 15-089 Bialystok, Poland; 4 Chair and Department of Oral Surgery, Medical University of Lublin, Doktora Witolda Chodźki 6 Street, 20-093 Lublin, Poland; 5 Department of Integrated Dentistry, Wroclaw Medical University, 50-425 Wroclaw, Poland

**Keywords:** anatomy, root resorption, retraction, bone density, orthodontic treatment, orthodontic diagnosis, periodontal ligament (PDL) stress, Finite Element Method (FEM)

## Abstract

Background: Hydrostatic pressure in the periodontal ligament (PDL) plays a critical role in orthodontic treatment, influencing tooth movement and remodeling of periodontal tissue. The relationship between alveolar cortical bone density and the risk of root resorption due to excessive stress in the PDL has not been clearly defined. Objective: This study aimed to analyze hydrostatic pressure in the periodontal ligament of the tooth roots during en-masse retraction of the maxillary incisors using temporary skeletal anchorage devices (TISADs) after the first premolar extractions, as well as during full arch retraction. Methods: A numerical model was used, varying the Young’s modulus of cortical bone from 12.5 GPa to 27.5 GPa in increments of 3.0 GPa. Extreme values for bone stiffness were derived from the literature. In all the cases analyzed, the hook height was fixed at 6 mm, and the cranial surface was constrained. Results: Doubling the stiffness of the cortical bone approximately reduced the hydrostatic pressure in the PDL by 1.5 times for both full-arch retraction and post-first premolar extraction retraction. A critical hydrostatic pressure of 4.7 kPa was exceeded in full-arch retraction for low Young’s modulus of 12.5 Gpa values at forces as low as 600 g. On the contrary, for cortical bone with a high Young’s modulus of 27.5 GPa, this critical pressure was reached only at forces around 960 g, approximately 1.6 times higher. Conclusions: The density of the alveolar cortical bone significantly influences the hydrostatic pressure in the PDL of most tooth roots during orthodontic treatment. This parameter can be a critical factor in the risk of root resorption when optimal forces are exceeded. Further research is necessary to better understand these dynamics. Individual protocols for orthodontic treatment and CBCT imaging are necessary to minimize complications in the form of root resorption.

## 1. Introduction

Hydrostatic pressure within the periodontal ligament (PDL) plays a critical role in orthodontic treatment, significantly affecting tooth movement and remodeling of periodontal tissues. When orthodontic forces are applied, PDL cells respond to altered mechanical conditions, resulting in localized changes in the pressure of the interstitial fluid. These variations can generate positive or negative stress, directly influencing bone resorption and deposition processes, which in turn determine the efficacy and stability of orthodontic therapy [1,2,3].

Bone mineral density (BMD) is a clinical parameter that is used to estimate bone mass, covering the total amount of bone matrix and minerals. These factors directly impact the mechanical strength of bone tissue. Bone density changes with age and is influenced by diet, smoking, genetic factors, and hormonal effects, especially estrogen in women. Systemic conditions such as diabetes, osteoporosis, and inflammatory gastrointestinal diseases also significantly affect BMD [4,5]. Furthermore, mechanical loading, muscle activity, and vitamin D intake positively influence bone strength and orthodontic stability, although vitamin D may slow tooth movement [6].

Various methods are available to assess bone density, including radiogrammetry, Compton scattering techniques, radiographic photodensitometry, single- or dual-energy photon absorptiometry, and quantitative computed tomography. A widely recognized classification by Lekholm and Zarb categorizes bone into four types (1–4) based on structural composition: Type 1 consists predominantly of compact bone; Type 2 features a thick layer of compact bone surrounding trabecular bone; Type 3 includes thin compact bone that envelopes dense trabecular bone; and Type 4, the weakest, comprises very thin compact bone surrounding sparse trabecular bone [7].

Most of the research focuses on the impact of bone density on the use of mini-implants in orthodontics, particularly on their placement, stability, load-bearing capacity, and risk of failure. Increased bone density is correlated with improved mechanical strength [8,9,10]. However, denser bone is associated with slower orthodontic tooth movement [11].

Hydrostatic pressure in the PDL, generated in response to orthodontic forces, is crucial to prevent root resorption caused by significant reductions or cessation of capillary blood flow. Pressure distribution depends on factors such as force magnitude, bracket slot size, archwire characteristics, and orthodontic techniques [12,13]. Currently, there is no clear relationship between alveolar cortical bone density and the risk of root resorption due to excessive stress in the PDL.

Direct measurement of PDL pressure and its distribution along root surfaces is challenging under clinical conditions. However, numerical analyses using finite element methods (FEM), introduced in orthodontics by Yettram et al. [14], provide insight into complex mechanical stress states in the maxilla, alveolar bone, and teeth. FEM simulations can identify specific loads and locations where pressure exceeds the vascular threshold in the PDL, potentially leading to complications such as root resorption.

Theoretical analyses suggest that the PDL pressure within a confined space is influenced by the mechanical properties of the surrounding walls, particularly the deformability of the alveolar bone. This deformability depends on bone density and other mechanical parameters. Identifying factors that influence PDL pressure could help classify patients at increased risk of complications such as root resorption and allow the development of targeted strategies to minimize these risks.

This study aimed to analyze the pressure of the PDL during en masse retraction of the maxillary incisors using temporary skeletal anchorage devices (TISADs) after the first premolar extractions and during full arch retraction. The analysis considered variations in alveolar cortical bone density using 0.016 × 0.022-inch and 0.017 × 0.025-inch stainless steel archwires in MBT brackets with a 0.018-slot, with a hook of 6 mm high.

The novelty of the article is the use of a numerical model that takes into account the exact structure of the skull base to analyze the effect of the density of the compacted bone on the load occurring in the PDL. The developed numerical model reproduces the geometry and strength properties of the teeth, PDL, cortical bone, cancellous bone, brackets, and arch. Furthermore, the numerical model incorporates not only the bone directly surrounding the PDL but also areas that extend significantly from it, which has a substantial impact on the results obtained.

There is no available research about the influence of bone density on PDL pressure and the risk of root resorption.

## 2. Materials and Methods

**Figure 1:**
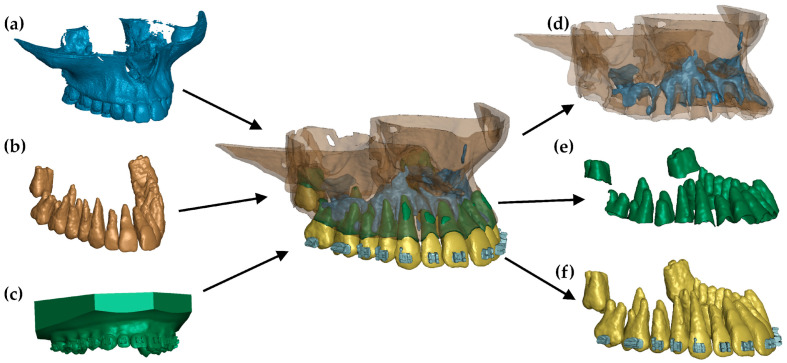
FE model: (**a**) geometry extracted from a CT image, (**b**) teeth extracted from a CT image, (**c**) scan of a dental arch with brackets, (**d**) combination of cortical and cancellous bone, (**e**) finite elements of the periodontium, and (**f**) teeth with brackets [12].

Bone strength increases with density. This study examined the influence of cortical bone stiffness on PDL pressure using a numerical model described in [12] (Figure 1). The model was developed based on a combination of head CT scans and intraoral teeth scans. The combination of these two sources of geometry made it possible to extract with very high accuracy: teeth, compact bone, spongy bone, and PDL. The geometry of the internal structures was extracted using MIMICS 18.0 software, where all structures were manually outlined on each DICOM image layer. A .stl file was then generated containing the closed outer surfaces of the internal structures and teeth. In the Hypermesh system, these outlines were filled with tetragonal (bones and teeth) and hexagonal (PDL) elements. In turn, the geometry of the brackets (which were modelled using tetragonal elements) and the arch (hexagonal elements) was determined from the 3D scans.

The properties of spongy bone, teeth, brackets, and arch were described using a linear elastic material with parameters described in [12]. The PDL was mapped using the Ogden model [12]. Young’s cortical bone modulus was varied between 12.5 GPa and 27.5 GPa in increments of 3.0 GPa, with extreme values derived from the literature [10,15]. A TIED-type contact was used between the structures that are connected (spongy bone, cortical bone, PDL, tooth, and brackets). A surface-to-surface contact based on a penalty function was defined between the brackets and the arch.

**Figure 2:**
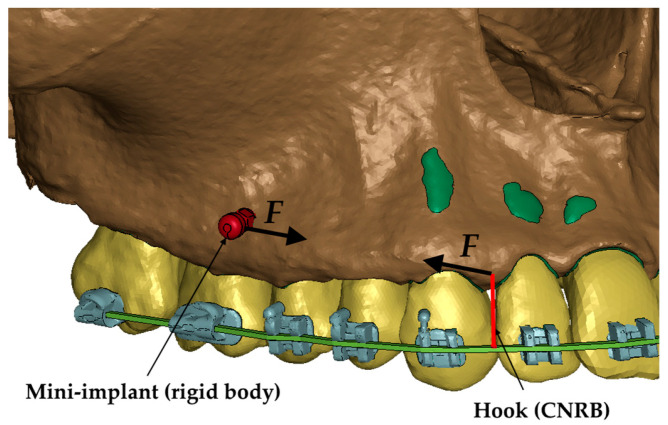
Load representation in the numerical model [12].

In all of the cases described in the paper, the load was defined between a 6 mm high hook attached to the arch and a mini-implant (Figure 2). The load ranged from 50 to 300 g. That force was chosen because the optimal translation movement due to Proffit should be made by 70–120 g [16]. In clinical practice, dynamometers are often not used, and the optimal force may be exceeded. However, the aim of the study is to determine whether and how the density of the alveolar bone plate affects the pressure in the periodontal ligament during axial movement.

The model was constrained by locking all degrees of freedom of the upper cranial surface.

Calculations were performed using an implicit time integration scheme in the LS-Dyna system. The applied load increased linearly from 0 at t(0) to a maximum value at t(1).

## 3. Results

**Figure 3:**
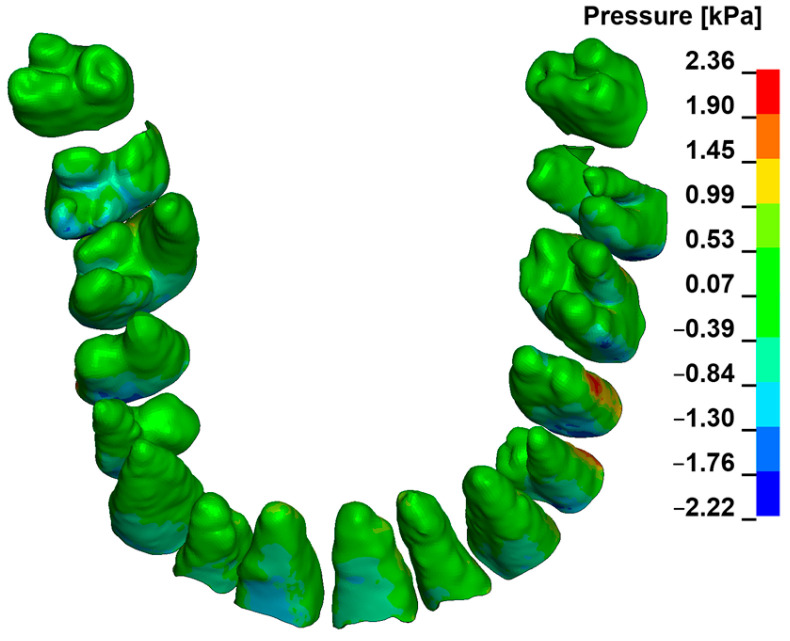
Maximal pressure value in the PDL for the entire dental arch.

**Figure 4:**
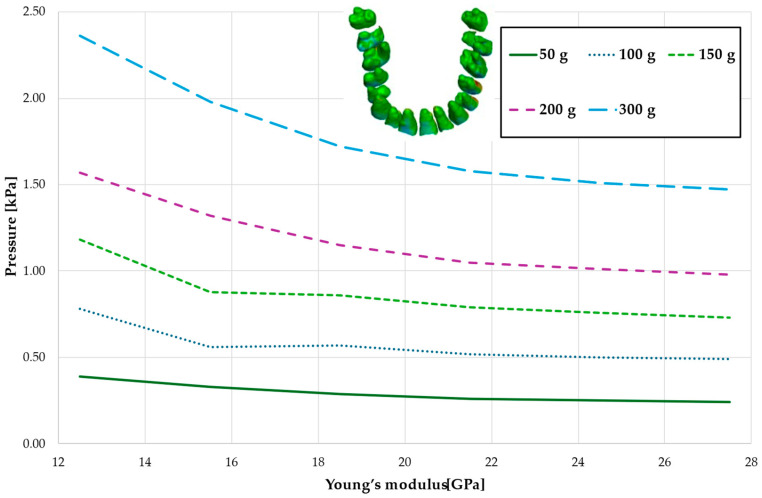
Maximum pressure value in the PDL for the entire dental arch.

**Figure 5:**
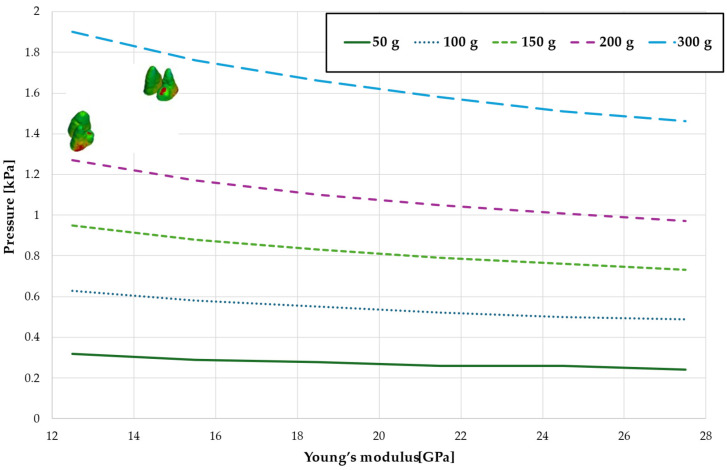
Maximal value of hydrostatic pressure in the periodontal ligament (PDL) surrounding the roots of the canines and first premolars.

**Figure 6:**
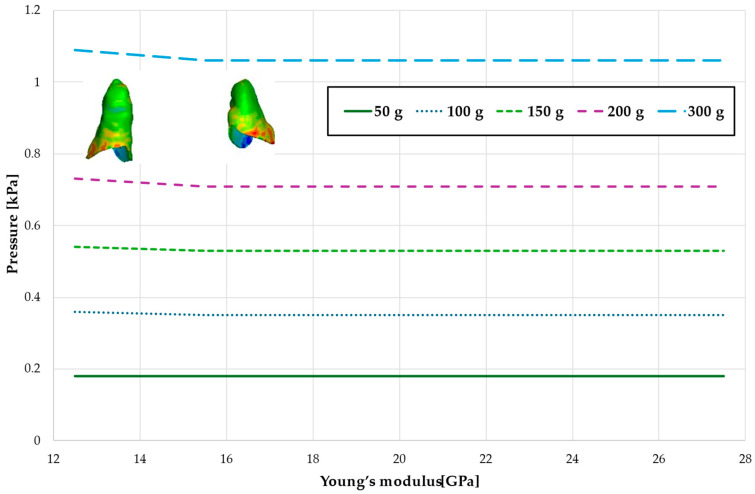
Maximal value of hydrostatic pressure in the periodontal ligament (PDL) surrounding the roots of the lateral incisors.

**Figure 7:**
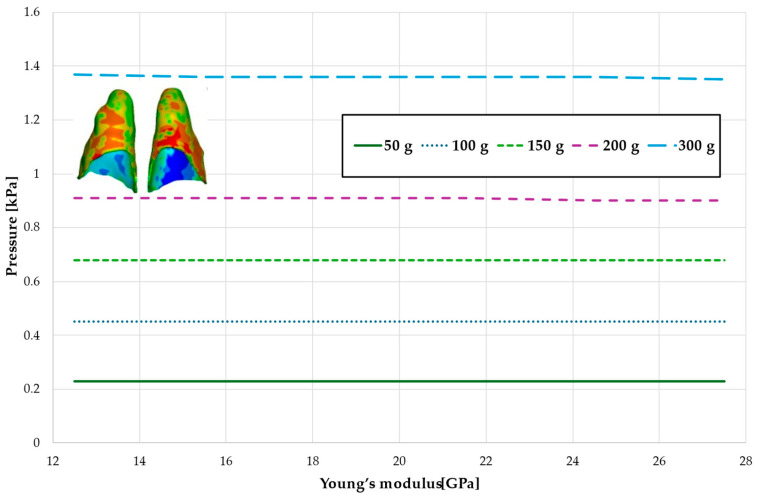
Maximal value of hydrostatic pressure in the periodontal ligament (PDL) surrounding the roots of the central incisors.

Figure 3, Figure 4, Figure 5, Figure 6 and Figure 7 present the maximum value of hydrostatic pressure in the periodontal ligament (PDL) during distalisation of the entire dental arch into orthodontic miniimplants placed buccally in the maxilla between the second premolar and the first molar. A 6 mm high hook was used in conjunction with a 0.017 × 0.025-inch stainless steel archwire in 0.018 slot brackets, applying orthodontic forces ranging from 50 to 300 g. Young’s modulus of the cortical alveolar bone ranged from 12.5 GPa to 27.5 GPa. Figure 3 presents the distribution of hydrostatic pressure in PDL for the Young’s modulus 12.5 GPa and 300 g load.

Figure 4 and Table 1 analyze the maximum pressure value in the PDL for the entire dental arch. In all cases, the hydrostatic pressure values decreased as the stiffness of the alveolar bone surrounding the tooth roots increased. The stiffer the alveolar bone, the stronger and more secure the tooth will be, as the increased bone rigidity provides better support. Approximately double the stiffness of the bone reduces the hydrostatic pressure by a factor of 1.5, thereby enhancing the resistance of the tooth to resorption and movement during orthodontic treatment.

Figure 5 and Table 2 present the maximum value of hydrostatic pressure in the periodontal ligament (PDL) surrounding the roots of the canines and the first premolars. A linear relationship is observed, where an increase in bone stiffness results in a reduction in hydrostatic pressure. Specifically, doubling the stiffness of the alveolar bone reduces the pressure by a factor of approximately 1.25.

Figure 6 and Figure 7 and Table 3 and Table 4 present the maximum value of hydrostatic pressure in the periodontal ligament (PDL) that surrounds the roots of the lateral and central incisors. In this case, bone density does not have a significant impact on measured values.

**Figure 8:**
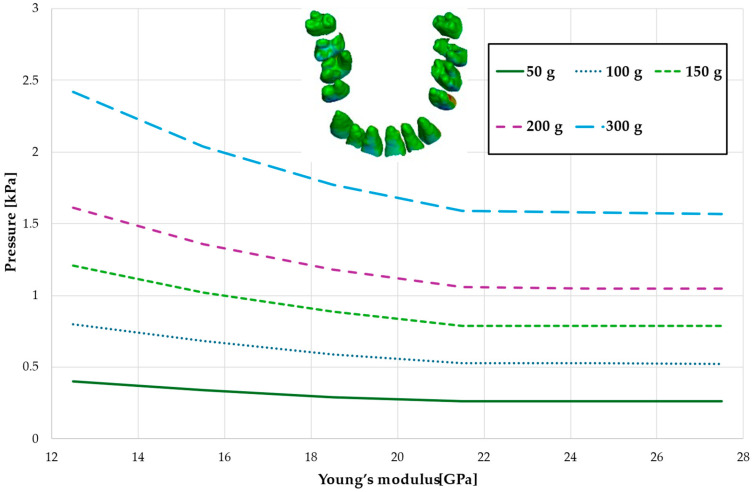
Maximal value of the pressure across the PDL of the entire dental arch.

**Figure 9:**
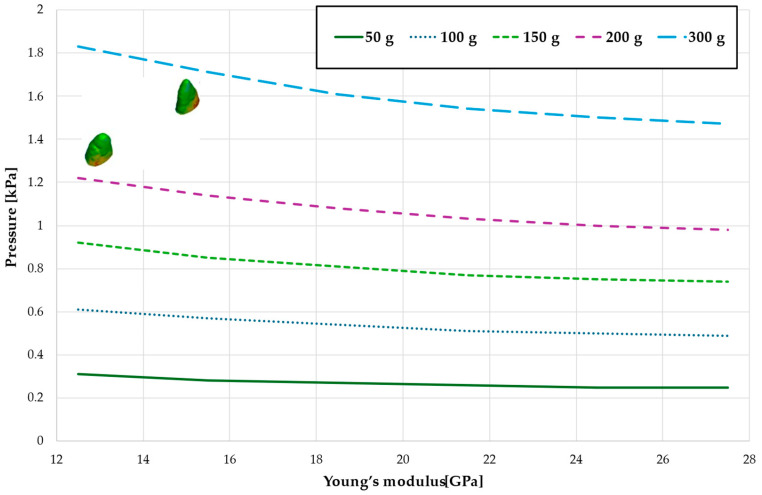
Maximal value of hydrostatic pressure in the periodontal ligament (PDL) surrounding the roots of the canines and first premolars.

**Figure 10:**
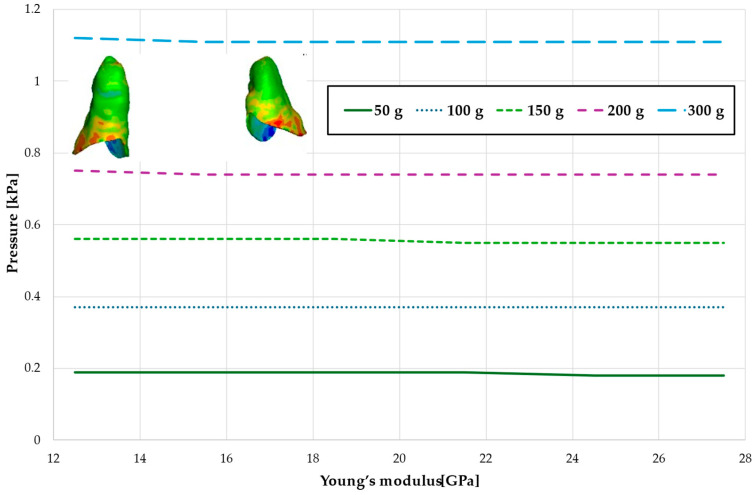
Maximal value of hydrostatic pressure in the periodontal ligament (PDL) surrounding the roots of the lateral incisors.

**Figure 11:**
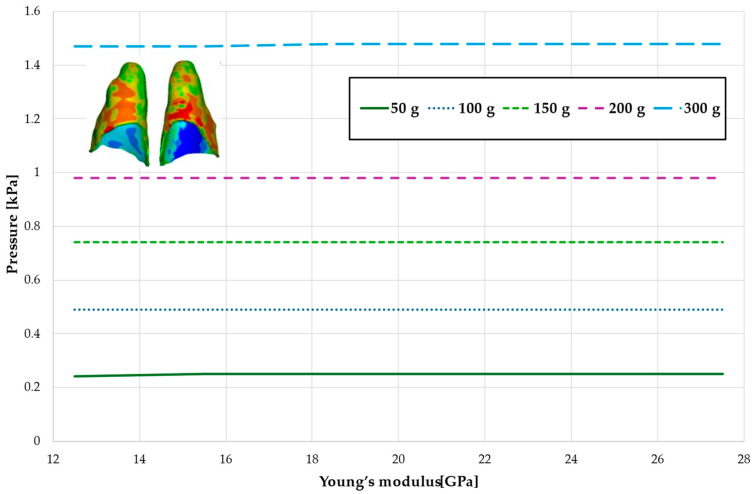
Maximal value of hydrostatic pressure in the periodontal ligament (PDL) surrounding the roots of the central incisors.

Figure 8, Figure 9, Figure 10 and Figure 11 and Table 5, Table 6, Table 7 and Table 8 illustrate the maximum values of hydrostatic pressure in the periodontal ligament (PDL) during the en masse retraction of upper anterior teeth after extraction of the first premolars. Orthodontic miniimplants were positioned vestibularly in the maxilla, between the second premolar and the first molar. A 6 mm hook was used in conjunction with a 0.017 × 0.025 stainless steel archwire and 0.018 slot brackets. Orthodontic forces ranging from 50 to 300 g were applied, with variations in Young’s modulus of the alveolar cortical bone of 12.5 to 27.5 GPa.

Figure 8 and Table 5 present a maximum pressure value throughout PDL of the entire dental arch. For higher values of bone stiffness (21.5–27.5 GPa), the applied orthodontic force generated similar PDL pressure levels. However, as the stiffness decreased, the pressure increased linearly, reaching approximately 1.5 times greater for the lowest stiffness value of 12.5 GPa.

Figure 9 and Table 6 present the hydrostatic pressure values in the periodontal ligament (PDL) around the roots of the canines. A similar trend is observed as previously described, with pressure decreasing as bone stiffness increases. However, the difference between the highest and lowest recorded pressures is approximately 1.24 times.

Figure 10 and Figure 11 and Table 7 and Table 8 present the hydrostatic pressure values in the periodontal ligament (PDL) around the roots of the lateral and central incisors. In these cases, bone density does not significantly influence pressure values. In all scenarios, the recorded pressures remain closely comparable.

**Figure 12:**
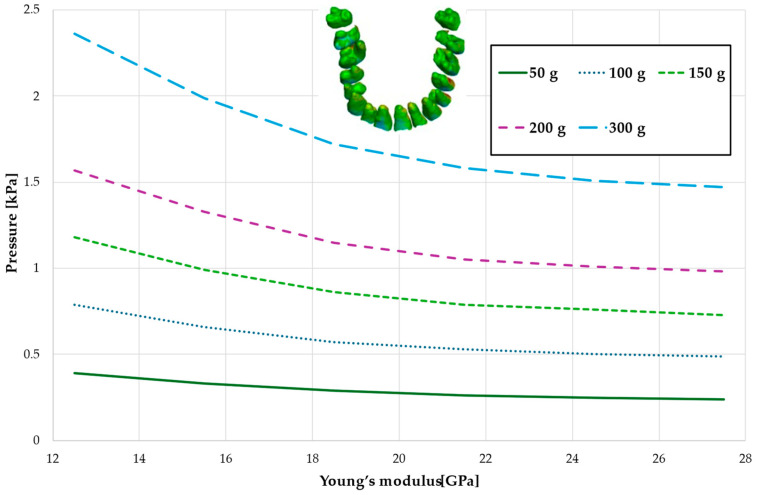
Maximum pressure value across the PDL of the entire dental arch.

**Figure 13:**
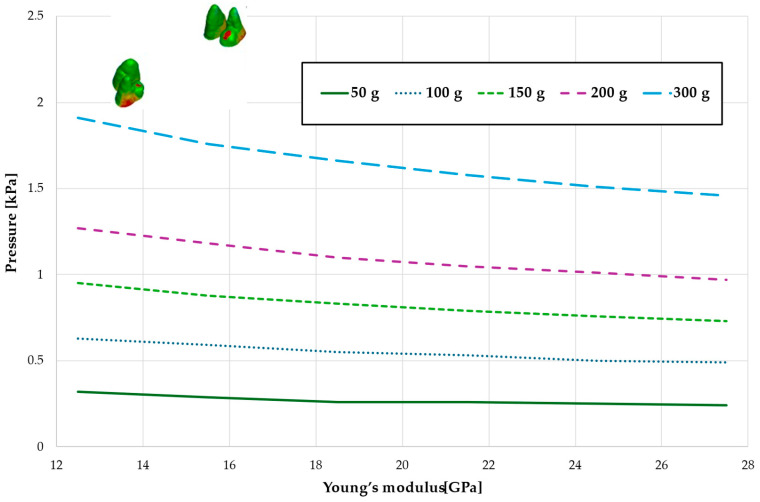
Maximal value of hydrostatic pressure in the periodontal ligament (PDL) surrounding the roots of the canines and first premolars.

**Figure 14:**
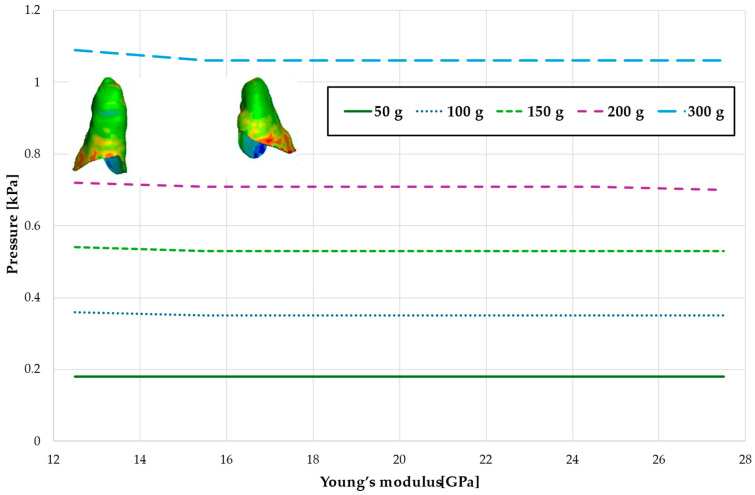
Maximal value of hydrostatic pressure in the periodontal ligament (PDL) surrounding the roots of the lateral incisors.

**Figure 15:**
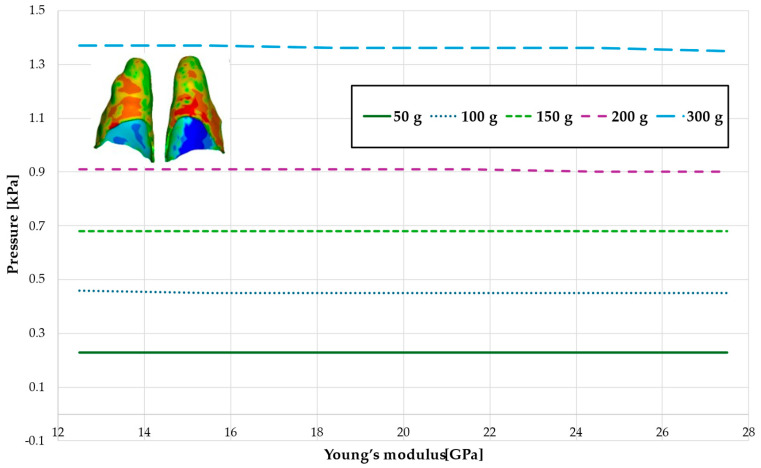
Maximal value of hydrostatic pressure in the periodontal ligament (PDL) surrounding the roots of the central incisors.

**Figure 16:**
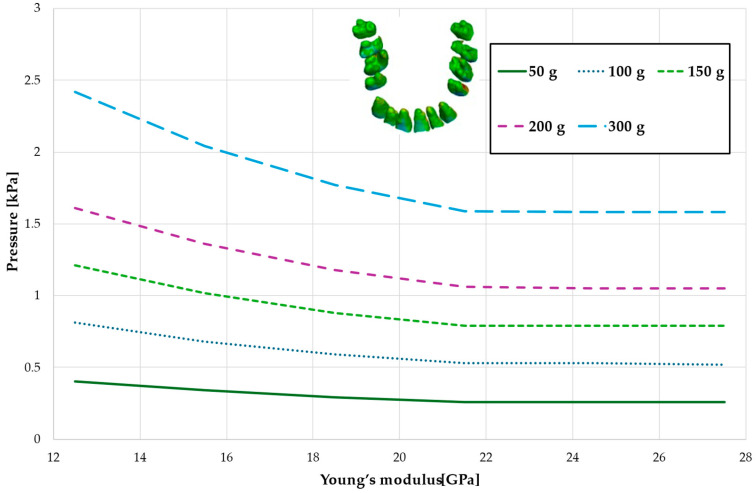
Maximal value of the pressure across the PDL of the entire dental arch.

**Figure 17:**
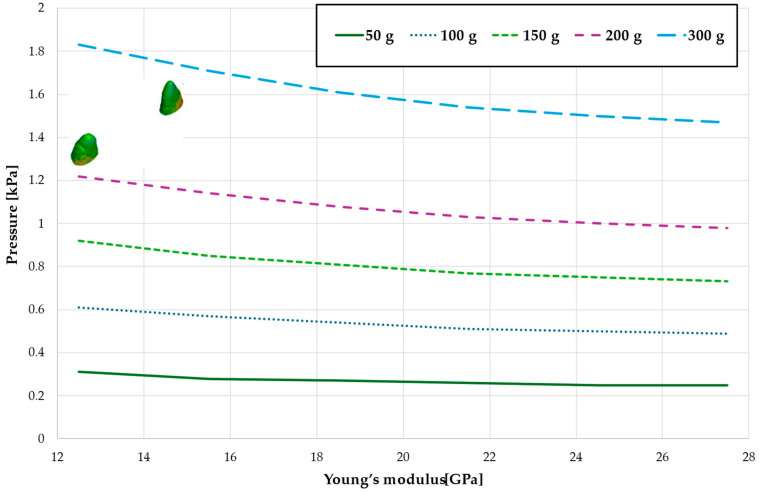
Maximal value of hydrostatic pressure in the periodontal ligament (PDL) surrounding the roots of the canines and first premolars.

**Figure 18:**
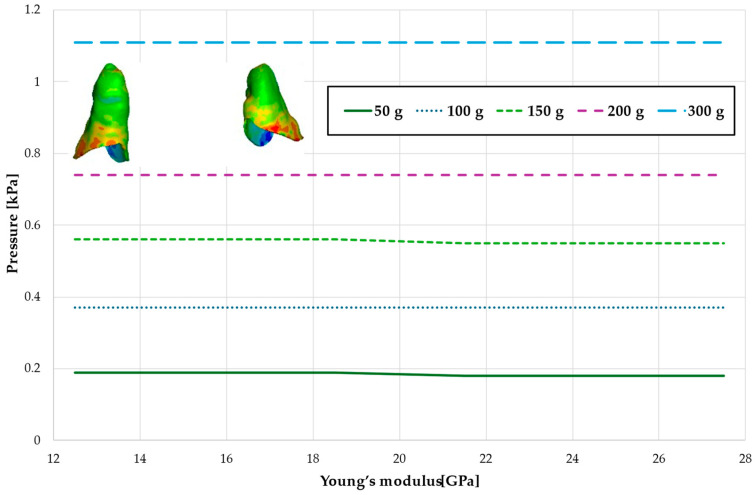
Maximal value of hydrostatic pressure in the periodontal ligament (PDL) surrounding the roots of the lateral incisors.

**Figure 19:**
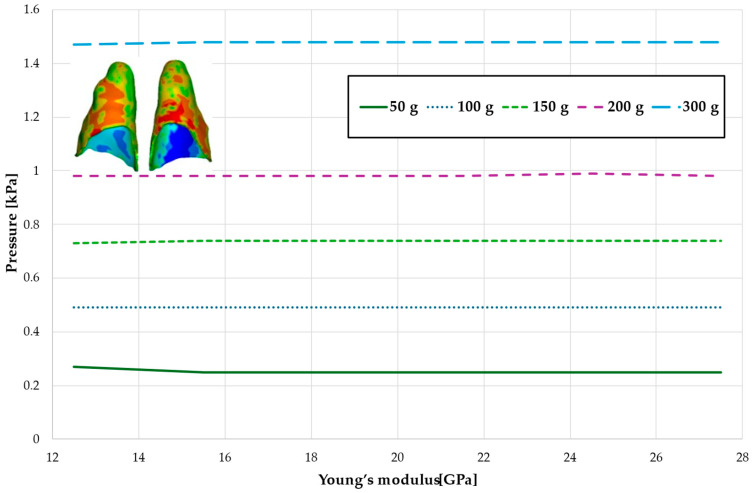
Maximal value of hydrostatic pressure in the periodontal ligament (PDL) surrounding the roots of the central incisors.

Figure 12, Figure 13, Figure 14 and Figure 15 and Table 9, Table 10, Table 11 and Table 12 present the results of full dental arch distalisation on a 0.016 × 0.022 steel arch with a 0.018 slot, while Figure 16, Figure 17, Figure 18 and Figure 19 and Table 13, Table 14, Table 15 and Table 16 illustrate en masse retraction using the same arch. The observed relationships follow similar patterns to those described previously.

On both arches, during full dental arch distalisation, the stress decreases linearly across the entire range of Young’s modulus variations. However, in the case of the first premolar extraction, the stress in the periodontal ligament decreases with increasing bone stiffness until it reaches an average value of approximately 21.5 GPa. Beyond this point, bone density no longer influences future changes in stress.

**Figure 20:**
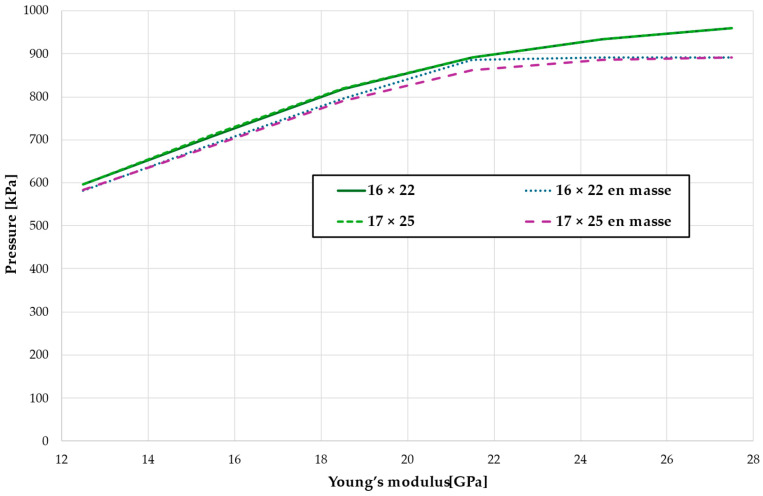
The load causing a critical value of 4.7 kPa in PDL.

The critical value of 4.7 kPa is exceeded for the entire dental arch with a low Young modulus at a force of approximately 600 g, while for bone with a high Young’s modulus value of 27.5 GPa, this threshold is reached only with a force of approximately 960 g, which is 1.6 times higher, as shown in Figure 20 and Table 17. The absence of first premolars slightly reduces the force required to reach the critical pressure in the periodontal ligament, but this effect is minimal, approximately 1.7% (see Figure 20).

**Figure 21:**
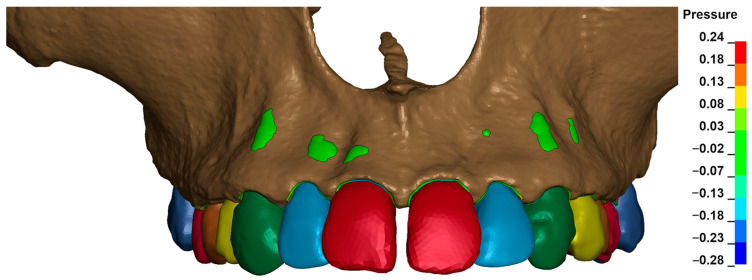

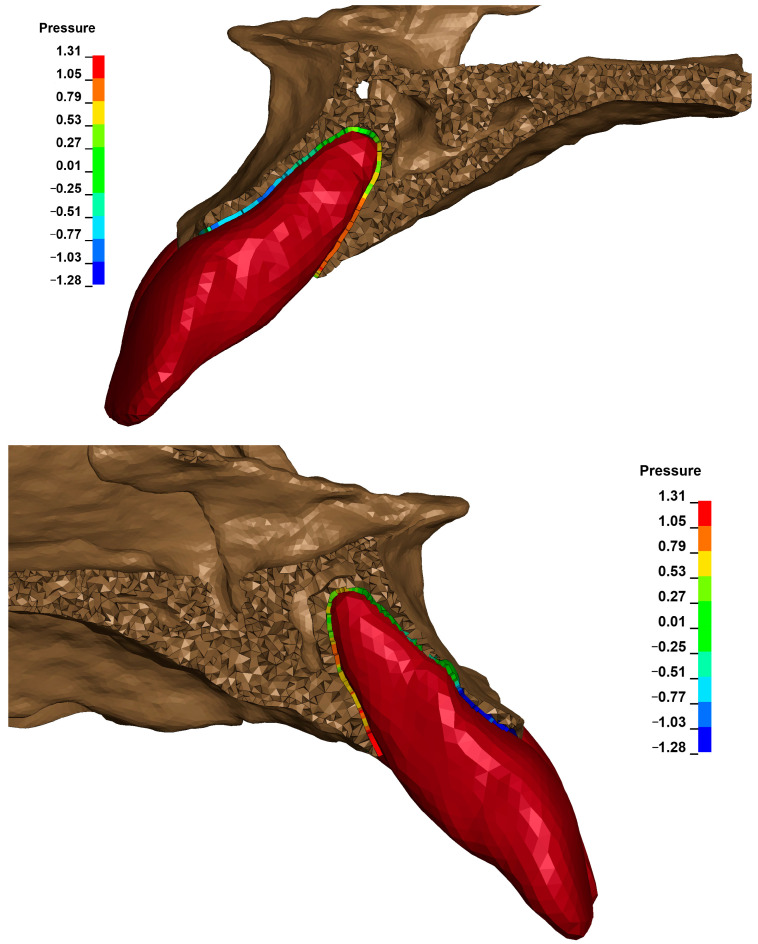
Cross section—the stress in the periodontal ligament of the upper central incisors during retraction.

The cross-sectional images shown in Figure 21 illustrate the stress in the periodontal ligament of the upper central incisors during retraction. On the left side, there is no fenestration of the bone, and the root is surrounded by cortical bone on all sides. On the right side, the presence of fenestration leads to reduced bone support, resulting in differentiated stress in the periodontal ligament, with higher stress values on the tooth with reduced support under the same orthodontic force.

**Figure 22:**
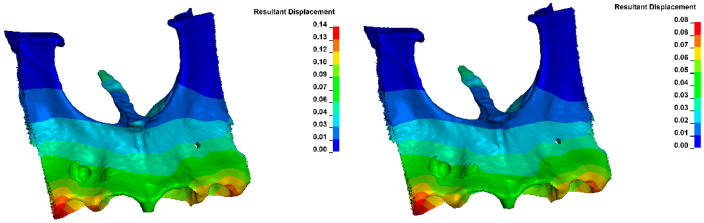
Displacement within the alveolus (for E = 12,400 MPa on the (**left**) and E = 27,500 MPa on the (**right**)).

Figure 22 shows the displacement within the alveolus after applying the same orthodontic force for different Young’s moduli. For E = 12,500 MPa, a displacement of about 0.14 μm was obtained, and for E = 27,500 MPa, about 0.08 μm.

## 4. Discussion

The periodontal ligament (PDL) is a tissue derived from cells originating in the dental follicle, differentiating into fibroblasts, cementoblasts, and osteoblasts. The bone surface is covered by osteoblasts, whereas the root surface is lined by cementoblasts, and fibroblasts form the periodontal ligaments, which consist mainly of collagen fibers and anchor the tooth within the alveolus. The PDL also contains numerous blood vessels [17]. Compression of these vessels, reducing blood flow or completely occluding their lumen, triggers a response from surrounding tissues to orthodontic tooth movement, leading to bone remodeling or hyalinisation of the compressed bone tissue. Stimulation of mechanosensitive ion channels and receptors in the cell membrane is induced by changes in cell shape due to the applied orthodontic forces [18]. Cells in the periodontium respond to mechanical stimuli through increased activity of cellular mediators, such as cyclic AMP (cAMP, cyclic adenosine monophosphate). This mediator activates protein kinases that catalyze the phosphorylation of proteins responsible for further signal transduction [19]. After several stages of signal processing, the signal reaches the cell nucleus, where two key processes are initiated: DNA (deoxyribonucleic acid) replication, leading to cell proliferation, and cell differentiation. Regulation involved in bone regeneration and cell adaptation occurs through phosphorylation of transcription factors such as c-Jun and c-Fos. Mediators play an important role, including protein kinase C (PKC) and pro-inflammatory compounds such as prostaglandin E2 (PGE2), which are released into the cytoplasm [20]. During inflammation, cyclooxygenase 2 (COX-2) is activated, leading to the formation of prostaglandin H2 (PGH2), which is then converted into PGE2 by prostaglandin E synthase. It has been demonstrated that PGE2, by interacting with E2 and E4 receptors, activates adenylate cyclase in PDL cells, contributing to increased bone resorption [21].

The periodontal ligament (PDL) is essential for orthodontic tooth movement. Consequently, a model should accurately simulate the movement of teeth through the periodontium and different layers of bone, using appropriate elastic moduli to ensure proper interaction. Previous research indicates that areas where the hydrostatic pressure (σ_h_) exceeds 4.7 kPa align with root resorption sites identified by electron microscopy. On the contrary, regions showing expansion in simulations did not show active resorption. Clinical investigations have similarly found no evidence of resorption defects in areas with high von Mises stress (σ_vM_) or minimum principal stress (σ_3_) values as determined through finite element analysis [12].

The results presented above are significant from both the perspective of the speed of orthodontic tooth movement and the prevention of unwanted orthodontically induced root resorption (OIRR). The goal of orthodontic treatment is to apply a force value that causes compression of blood vessels in the PDL, thus initiating a molecular mechanism leading to bone remodeling. According to the literature, the optimal force level for distalization or retraction is approximately 120–150 g for all patients [16]. However, it appears that such generalization can increase the risk of resorption and slow tooth movement in certain patients, depending on the bone density of the alveolar process. For many years, the topic of optimal loading of the periodontal ligament during orthodontic treatment has been highly controversial due to several factors. The first challenge is the inability to accurately determine the distribution of stresses and strains within the periodontal ligament. Another problem is that many studies have not considered the type of tooth movement performed. In most cases, tipping movements were studied, leading to uneven stress and strain distribution in the periodontal ligament. An additional difficulty in analyzing the relationship between force and tooth movement speed is the division of orthodontic tooth movement into several phases. Ultimately, both human and animal studies have shown considerable individual variability. Even when uniform, equal forces are applied, the rate of tooth movement can vary significantly between individuals and even within the same individual [22].

In general, higher levels of pressure are applied when a force is applied to a tooth whose root is anchored in a space surrounded by a wall of material with greater stiffness. However, the studies described above clearly indicate that the same orthodontic force applied to the teeth of patients with low bone stiffness results in 1.5 to 1.6 times higher stress in PDL than in individuals with dense and well-mineralized bone tissue. Patients with low Young’s modulus typically include younger people with open bite configurations, coexisting with loose masticatory muscles [23], people with reduced physical activity, people with osteoporosis [24], and people with low levels of vitamin D [25].

Individuals with low estrogen levels [26] or those with low bone density, such as young people with an open bite configuration or those with weak masticatory muscles, typically exhibit low bone mineralization, resulting in a low Young’s modulus. On the contrary, individuals with a deep bite configuration and overactive masticatory muscles are characterized by high bone mineralization or a high Young’s modulus [23]. As camouflage treatments involving extraction of first premolars and retraction of incisors are more commonly performed in the first group, it is crucial to recognize that orthodontic forces for these patients should be applied with caution, at values approximately 1.5 times smaller than the generally accepted optimal forces, in order to achieve a similar response from periodontal tissue.

Elastic chains can be particularly risky for these individuals, as they initially generate maximum force after placement, which can exceed the critical threshold. Before the force decreases, this could initiate hyalinisation, slowing tooth movement and leading to undesirable root resorption. The Double Unitek AlastiK C2 links, after being stretched by 17 mm, demonstrated an initial force of approximately 641 g (22.5 ounces), which is higher than the average of 342 g (12.0 ounces) generated by Ormco Power Chain. Within one day, the force was reduced to 171 g (6.0 ounces) for both materials [27]. The force of 641 g can already cause complete occlusion of the blood vessels, according to our findings [12].

A more reasonable alternative for these patients appears to be the use of NiTi (nickel-titanium) springs, which generate a more consistent force. Studies have shown that these springs experienced a statistically significant force reduction of approximately 12% after 4 weeks of clinical use (*p* < 0.01), followed by a further decrease of approximately 7% between the 4th and 8th week (*p* = 0.03), after which the force remained stable. Despite this reduction, the closure of the space continued at a rate of approximately 0.91 mm per month. Analysis of force loss in both in vivo and in vitro conditions revealed no significant differences. Nickel-titanium springs with closed coils, although they do not maintain a constant force in the intraoral environment, still enable effective space closure at a rate of approximately 1 mm per month [28].

It is also important to note that this statement does not refer to the total force value but rather to the force applied per unit surface area of the periodontal ligament. This distinction is crucial because reduced bone density also affects individuals with reduced periodontal tissue and, therefore, a smaller surface area [29]. On the contrary, in the present study, the surface area of the periodontal ligament remained unchanged, and only the bone density of the alveolar process varied. High bone density generates greater stiffness and resistance, thereby reducing the amount of force transmitted to the most stressed anterior teeth. In cases of low bone density, reduced resistance allows for greater bone deflection and easier compliance of the alveolar bone, resulting in a more significant displacement of the anterior teeth. Consequently, the same force generates greater displacement in the alveolus, increasing the likelihood of complete occlusion of the blood vessels and, ultimately, necrosis and atrophy of the surrounding tissues.

The displacement within the alveolus, after the application of the same orthodontic force, varies depending on bone density, as shown in Figure 22. The displacement is 1.75 times greater with a low Young modulus than with a high modulus.

It can be illustrated that the alveolar bone plate acts as a protective shield for the root of the tooth. The thicker the cortical bone, the more secure and resistant the tooth is to resorption. Interestingly, the most heavily stressed areas in all situations are the distal-labial surfaces of the canines and, possibly, the first premolars.

Another notable observation is that with the 0.016 × 0.022 steel arch, it is easier to induce the twisting of the arch in the brackets due to the force applied to the hook. This results in proclination of the incisors, masking less torque control than in the 0.017 × 0.025 arch. This leads to similar stress on the anterior teeth.

A key finding of the research is that the pressure on the periodontal ligament on the palatal side is highly dependent on the thickness and presence of the vestibular cortical plate. This plate acts as resistance and counterbalance when an orthodontic force is applied. When present, the cortical plate in the palatal aspect results in lower negative stress and less stretching on the vestibular side. In cases of localized fenestration, the same force generates significantly greater pressure on the palatal side and reduced resistance on the vestibular side, allowing greater displacement and an increased risk of resorption.

It can be hypothesized that excessive expansion of the dental arch, which leads to thinning and possible dehiscence of the alveolar process, increases the risk of resorption when torque is applied to excessively proclined teeth. This is due to both increased stress in the periodontal ligament and the lack of resistance on the vestibular side, as well as the contact between the roots and the cortical plate. Similarly, in the cases of advanced incisor protrusion, with a thin layer of bone over the vestibular aspect, these mechanisms drastically increase the risk of resorption. In such cases, it is recommended to apply at least 1.5–1.7 times smaller forces for tipping and retraction. In addition, a corticotomy can be considered to support treatment. The impact on periodontal tissues remains controversial due to the low quality of available studies [30], although long-term observations are promising [31,32].

The research presented in the article has some limitations. The numerical model is based on a CT scan of a specific patient, which means that it reproduces the geometric structure and the distribution between the individual compact and cortical bone of that patient. Another patient will have a different geometry and a different percentage distribution of compact and cortical bone. The results presented here, therefore, show the change in load in the PDL as a function of bone density for that particular patient. However, the trend of these changes is common to all patients and will vary over the range of values.

The specific value of a patient’s Young’s modulus depends on bone density and individual biological conditions, which are difficult to estimate from CT scans alone. The study adopted values of material from the literature. Determining them precisely would require invasive testing, which is currently not possible. However, technical solutions are being developed to overcome this limitation, such as the Simdencisty System.

This method does not permit the examination of how gender, age, and other systemic factors affect the periodontal response to orthodontic force. It also does not allow for the investigation of how these factors may interfere with the molecular mechanism in response to pressure or total vascular occlusion.

The systematic review of Dibello concluded that the number of teeth showed a negative correlation with fractures and reduced bone mineral density, while periodontal disease was positively linked to osteoporosis and lower bone mineral density. Masticatory function was associated only with osteoporosis, overall oral health was related solely to fractures, and occlusal force was connected exclusively to bone mineral density. Among oral health indicators, the number of teeth was the most frequently associated with bone mineral density disorders. These findings may help evaluate the impact of various oral health factors on the development of bone mineral density disorders in older age [33]. Diabetes can reduce bone density as well [34]. Another research found a positive correlation between HDL-C levels and lumbar BMD in individuals aged 20 to 59. These findings suggest that HDL-C measurement could serve as a useful biomarker for early osteoporosis detection, less bone density, and treatment guidance [35].

## 5. Conclusions

The density of the compact alveolar bone plate has a significant effect on the hydrostatic pressure value in the periodontal ligament of most tooth roots during orthodontic treatment.

When optimal forces of 120 to 150 g per side are applied, no risk of root resorption is observed due to the even distribution of light and moderate stress within the examined bone stiffness values.

The application of 600 g orthodontic forces, such as those generated by elastic chains, can initiate the resorption process by closing the capillaries. The risk of this increases in patients with low bone stiffness.

The presence or absence of first premolars does not affect the threshold of forces that exceed 4.7 kPa, which depends on Young’s modulus of the compact alveolar bone plate. A stiffer bone plate offers greater protection against hyalinization and slows tooth movement.

Individual protocols for orthodontic treatment and CBCT imaging are necessary to minimize complications in the form of root resorption.

Non-linear models are recommended for FEA analysis to ensure the reliability, comparability, and close approximation of results to the conditions found in the oral cavity.

In the next stages of the work, the authors will aim to develop a method to validate the numerical model described in the paper. Further work focused on modeling and analyzing the effect of bone density on loading in PDL is also planned using different patient cases. This will allow the results to be extended to include cases with different biological conditions, genders, and ages.

## Figures and Tables

**Table 1:** Maximum pressure value in the PDL for the entire dental arch.

Load [g]	Young’s Modulus [GPa]					
	12.5	15.5	18.5	21.5	24.5	27.5
50	0.39	0.33	0.29	0.26	0.25	0.24
100	0.78	0.56	0.57	0.52	0.50	0.49
150	1.18	0.88	0.86	0.79	0.76	0.73
200	1.57	1.32	1.15	1.05	1.01	0.98
300	2.36	1.98	1.72	1.58	1.51	1.47

**Table 2:** Maximal value of hydrostatic pressure in the periodontal ligament (PDL) surrounding the roots of the canines and first premolars.

Load [g]	Young’s Modulus [GPa]					
	12.5	15.5	18.5	21.5	24.5	27.5
50	0.32	0.29	0.28	0.26	0.26	0.24
100	0.63	0.58	0.55	0.52	0.50	0.49
150	0.95	0.88	0.83	0.79	0.76	0.73
200	1.27	1.17	1.10	1.05	1.01	0.97
300	1.90	1.76	1.66	1.58	1.51	1.46

**Table 3:** Maximal value of hydrostatic pressure in the periodontal ligament (PDL) surrounding the roots of the lateral incisors.

Load [g]	Young’s Modulus [GPa]					
	12.5	15.5	18.5	21.5	24.5	27.5
50	0.18	0.18	0.18	0.18	0.18	0.18
100	0.36	0.35	0.35	0.35	0.35	0.35
150	0.54	0.53	0.53	0.53	0.53	0.53
200	0.73	0.71	0.71	0.71	0.71	0.71
300	1.09	1.06	1.06	1.06	1.06	1.06

**Table 4:** Maximal value of hydrostatic pressure in the periodontal ligament (PDL) surrounding the roots of the central incisors.

Load [g]	Young’s Modulus [GPa]					
	12.5	15.5	18.5	21.5	24.5	27.5
50	0.23	0.23	0.23	0.23	0.23	0.23
100	0.45	0.45	0.45	0.45	0.45	0.45
150	0.68	0.68	0.68	0.68	0.68	0.68
200	0.91	0.91	0.91	0.91	0.90	0.90
300	1.37	1.36	1.36	1.36	1.36	1.35

**Table 5:** Maximal value of the pressure across the PDL of the entire dental arch.

Load [g]	Young’s Modulus [GPa]					
	12.5	15.5	18.5	21.5	24.5	27.5
50	0.40	0.34	0.29	0.26	0.26	0.26
100	0.80	0.68	0.59	0.53	0.53	0.52
150	1.21	1.02	0.89	0.79	0.79	0.79
200	1.61	1.36	1.18	1.06	1.05	1.05
300	2.42	2.04	1.77	1.59	1.58	1.57

**Table 6:** Maximal value of hydrostatic pressure in the periodontal ligament (PDL) surrounding the roots of the canines and first premolars.

Load [g]	Young’s Modulus [GPa]					
	12.5	15.5	18.5	21.5	24.5	27.5
50	0.31	0.28	0.27	0.26	0.25	0.25
100	0.61	0.57	0.54	0.51	0.50	0.49
150	0.92	0.85	0.81	0.77	0.75	0.74
200	1.22	1.14	1.08	1.03	1.00	0.98
300	1.83	1.71	1.61	1.54	1.50	1.47

**Table 7:** Maximal value of hydrostatic pressure in the periodontal ligament (PDL) surrounding the roots of the lateral incisors.

Load [g]	Young’s Modulus [GPa]					
	12.5	15.5	18.5	21.5	24.5	27.5
50	0.19	0.19	0.19	0.19	0.18	0.18
100	0.37	0.37	0.37	0.37	0.37	0.37
150	0.56	0.56	0.56	0.55	0.55	0.55
200	0.75	0.74	0.74	0.74	0.74	0.74
300	1.12	1.11	1.11	1.11	1.11	1.11

**Table 8:** Maximal value of hydrostatic pressure in the periodontal ligament (PDL) surrounding the roots of the central incisors.

Load [g]	Young’s Modulus [GPa]					
	12.5	15.5	18.5	21.5	24.5	27.5
50	0.24	0.25	0.25	0.25	0.25	0.25
100	0.49	0.49	0.49	0.49	0.49	0.49
150	0.74	0.74	0.74	0.74	0.74	0.74
200	0.98	0.98	0.98	0.98	0.98	0.98
300	1.47	1.47	1.48	1.48	1.48	1.48

**Table 9:** Maximum pressure value across the PDL of the entire dental arch.

Load [g]	Young’s Modulus [GPa]					
	12.5	15.5	18.5	21.5	24.5	27.5
50	0.39	0.33	0.29	0.26	0.25	0.24
100	0.79	0.66	0.57	0.53	0.5	0.49
150	1.18	0.99	0.86	0.79	0.76	0.73
200	1.57	1.33	1.15	1.05	1.01	0.98
300	2.36	1.99	1.72	1.58	1.51	1.47

**Table 10:** Maximal value of hydrostatic pressure in the periodontal ligament (PDL) surrounding the roots of the canines and first premolars.

Load [g]	Young’s Modulus [GPa]					
	12.5	15.5	18.5	21.5	24.5	27.5
50	0.32	0.29	0.26	0.26	0.25	0.24
100	0.63	0.59	0.55	0.53	0.50	0.49
150	0.95	0.88	0.83	0.79	0.76	0.73
200	1.27	1.18	1.10	1.05	1.01	0.97
300	1.91	1.76	1.66	1.58	1.51	1.46

**Table 11:** Maximal value of hydrostatic pressure in the periodontal ligament (PDL) surrounding the roots of the lateral incisors.

Load [g]	Young’s Modulus [GPa]					
	12.5	15.5	18.5	21.5	24.5	27.5
50	0.18	0.18	0.18	0.18	0.18	0.18
100	0.36	0.35	0.35	0.35	0.35	0.35
150	0.54	0.53	0.53	0.53	0.53	0.53
200	0.72	0.71	0.71	0.71	0.71	0.70
300	1.09	1.06	1.06	1.06	1.06	1.06

**Table 12:** Maximal value of hydrostatic pressure in the periodontal ligament (PDL) surrounding the roots of the central incisors.

Load [g]	Young’s Modulus [GPa]					
	12.5	15.5	18.5	21.5	24.5	27.5
50	0.23	0.23	0.23	0.23	0.23	0.23
100	0.46	0.45	0.45	0.45	0.45	0.45
150	0.68	0.68	0.68	0.68	0.68	0.68
200	0.91	0.91	0.91	0.91	0.90	0.90
300	1.37	1.37	1.36	1.36	1.36	1.35

**Table 13:** Maximal value of the pressure across the PDL of the entire dental arch.

Load [g]	Young’s Modulus [GPa]					
	12.5	15.5	18.5	21.5	24.5	27.5
50	0.40	0.34	0.29	0.26	0.26	0.26
100	0.81	0.68	0.59	0.53	0.53	0.52
150	1.21	1.02	0.88	0.79	0.79	0.79
200	1.61	1.36	1.18	1.06	1.05	1.05
300	2.42	2.04	1.77	1.59	1.58	1.58

**Table 14:** Maximal value of hydrostatic pressure in the periodontal ligament (PDL) surrounding the roots of the canines and first premolars.

Load [g]	Young’s Modulus [GPa]					
	12.5	15.5	18.5	21.5	24.5	27.5
50	0.31	0.28	0.27	0.26	0.25	0.25
100	0.61	0.57	0.54	0.51	0.50	0.49
150	0.92	0.85	0.81	0.77	0.75	0.73
200	1.22	1.14	1.08	1.03	1.00	0.98
300	1.83	1.71	1.61	1.54	1.50	1.47

**Table 15:** Maximal value of hydrostatic pressure in the periodontal ligament (PDL) surrounding the roots of the lateral incisors.

Load [g]	Young’s Modulus [GPa]					
	12.5	15.5	18.5	21.5	24.5	27.5
50	0.19	0.19	0.19	0.18	0.18	0.18
100	0.37	0.37	0.37	0.37	0.37	0.37
150	0.56	0.56	0.56	0.55	0.55	0.55
200	0.74	0.74	0.74	0.74	0.74	0.74
300	1.11	1.11	1.11	1.11	1.11	1.11

**Table 16:** Maximal value of hydrostatic pressure in the periodontal ligament (PDL) surrounding the roots of the central incisors.

Load [g]	Young’s Modulus [GPa]					
	12.5	15.5	18.5	21.5	24.5	27.5
50	0.27	0.25	0.25	0.25	0.25	0.25
100	0.49	0.49	0.49	0.49	0.49	0.49
150	0.73	0.74	0.74	0.74	0.74	0.74
200	0.98	0.98	0.98	0.98	0.99	0.98
300	1.47	1.48	1.48	1.48	1.48	1.48

**Table 17:** The load [g] causing a critical value of 4.7 kPa in PDL.

Configuration	Young’s Modulus [GPa]					
	12.5	15.5	18.5	21.5	24.5	27.5
16 × 22	597	708	818	892	934	959
16 × 22 en masse	582	691	796	886	892	892
17 × 25	597	712	819	892	933	959
17 × 25 en masse	583	686	790	862	886	892

## Data Availability

The original contributions presented in this study are included in the article. Further inquiries can be directed to the corresponding author.
